# Involvement of the GH38 Family Exoglycosidase α-Mannosidase in Strawberry Fruit Ripening

**DOI:** 10.3390/ijms25126581

**Published:** 2024-06-14

**Authors:** Angela Méndez-Yáñez, Darwin Sáez, Francisca Rodríguez-Arriaza, Claudio Letelier-Naritelli, Felipe Valenzuela-Riffo, Luis Morales-Quintana

**Affiliations:** 1 Multidisciplinary Agroindustry Research Laboratory, Instituto de Ciencias Biomédicas, Facultad de Ciencias de la Salud, Universidad Autónoma de Chile, Cinco Poniente #1670, Talca 3467987, Chile; 2 Programa de Doctorado en Ciencias Biomédicas, Instituto de Ciencias Biomédicas, Facultad de Ciencias de la Salud, Universidad Autónoma de Chile, Cinco Poniente #1670, Talca 3467987, Chile; 3 Instituto de Ciencias Biológicas, Universidad de Talca, Campus Talca, Avenida Lircay s/n, Talca 3460000, Chile

**Keywords:** α-mannosidase, exoglycosidase, *Fragaria* × *ananassa*, fruit ripening, N-glycosylation

## Abstract

Exoglycosidase enzymes hydrolyze the N-glycosylations of cell wall enzymes, releasing N-glycans that act as signal molecules and promote fruit ripening. Vesicular exoglycosidase α-mannosidase enzymes of the GH38 family (EC 3.2.1.24; α-man) hydrolyze N-glycans in non-reduced termini. Strawberry fruit (*Fragaria* × *ananassa*) is characterized by rapid softening as a result of cell wall modifications during the fruit ripening process. Enzymes acting on cell wall polysaccharides explain the changes in fruit firmness, but α-man has not yet been described in *F.* × *ananassa*, meaning that the indirect effects of N-glycan removal on its fruit ripening process are unknown. The present study identified 10 GH38 α-man sequences in the *F.* × *ananassa* genome with characteristic conserved domains and key residues. A phylogenetic tree built with the neighbor-joining method and three groups of α-man established, of which group I was classified into three subgroups and group III contained only *Poaceae* spp. sequences. The real-time qPCR results demonstrated that *FaMAN* genes decreased during fruit ripening, a trend mirrored by the total enzyme activity from the white to ripe stages. The analysis of the promoter regions of these *FaMAN* genes was enriched with ripening and phytohormone response elements, and contained *cis*-regulatory elements related to stress responses to low temperature, drought, defense, and salt stress. This study discusses the relevance of α-man in fruit ripening and how it can be a useful target to prolong fruit shelf life.

## 1. Introduction

In plants, approximately half of all proteins undergo N-glycosylation [1]. These post-translational modifications (PTMs) include the attachment of glycans, forming complex sugar structures interconnected via glycosidic bonds, initially with a glycan bonded covalently to an ASN residue [2]. This process has been extensively detailed in previous studies, emphasizing the involvement of specific enzymes [3,4,5]. Despite limited exploration, the impact of N-glycosylation on plant proteins and enzymes is notable; its absence has been associated with changes in biochemical properties, such as decreased stability, thermostability, and function in N-glycoproteins, with differential relative expression patterns during the development and fruit ripening stages [2,6,7,8].

During fruit ripening, crucial organoleptic features, such as aroma, antioxidant capacity, and texture, undergo alterations, significantly influencing the overall quality and consumer acceptance of the produce [9]. Fruit firmness is closely linked to ripening and alterations in texture, during which cell wall proteins (CWPs) cleave and modify the polysaccharides of the cell wall [10,11,12]. Furthermore, the N-glycans released from N-glycosylations promote fruit ripening [13]. The cleavage of N-glycans is carried out by de-N-glycosyl enzymes, which can split N-glycan structures both internally and externally [14]. The structures of N-glycosylation undergo changes according to tissue specificity and environmental conditions, resulting in variations in the N-glycosylation patterns of proteins. Additionally, a specific N-glycosylation site may undergo glycosylation during one stage and exhibit alterations in subsequent stages [2]. α-D-mannosidase mannohydrolase (GH38 family; EC 3.2.1.24; α-man) is an exoglycosidase with activity on α-1,2-, α-1,3-, and α-1,6-linked non-reducing α-D-mannose N-glycans in α-D-mannosides [15]. The suppression of α-man extends the shelf life of fruit by ~30 days in *Solanum lycopersicum* and ~7 days in *Capsicum annuum* [16,17]. Throughout development and fruit ripening, the enzyme activity of α-man increases in *C. annuum* and *Prunus salicina* [17,18,19], but decreases in *Pyrus communis*, *S. lycopersicum*, *Olea europea*, and *C. annuum* ‘Variata’ [16,20,21]. In *F.* × *ananassa*, a fruit characterized by its short shelf life, previous research has shown elevated enzyme activity levels and an increased relative expression of α-man postharvest in fruit treated with alginate [22]. However, there are scarce information about α -man in commercial strawberries and it is also unknown whether this enzyme is indirectly involved in ripening. Consequently, our objective was to contribute to the understanding of fruit ripening by investigating the role of α-man in *F.* × *ananassa* ‘Camarosa’, a cultivar known for its firmer texture [23]. We initially assessed the in silico parameters of the identified isozymes and carried out real-time qPCR and enzyme activity assays to elucidate the indirect relationship of α-man in fruit development and ripening.

## 2. Results and Discussion

### 2.1. Computational Analysis

#### 2.1.1. Candidate Genes of α-Man from *F.* × *ananassa*

**Figure 1:**
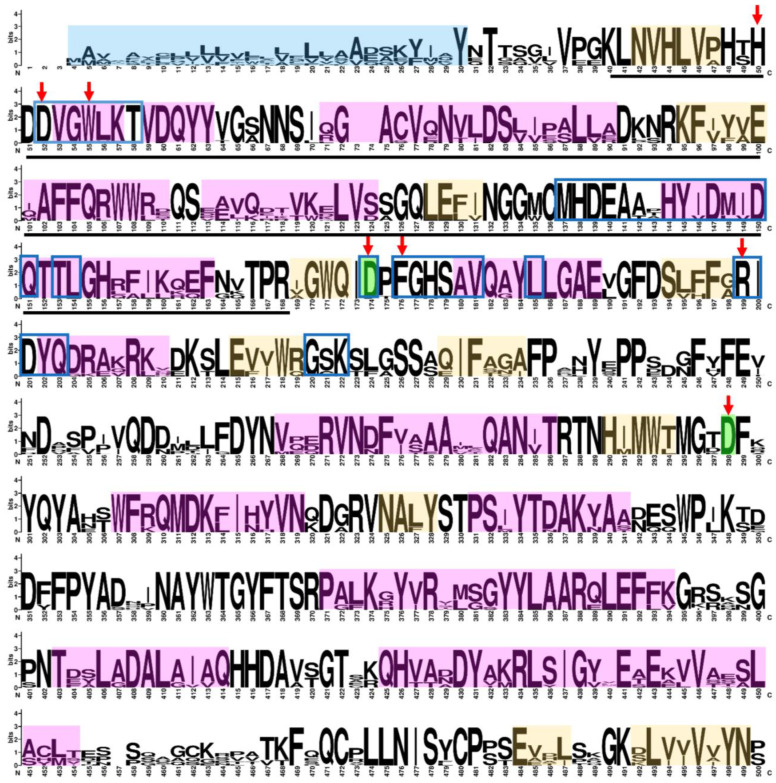

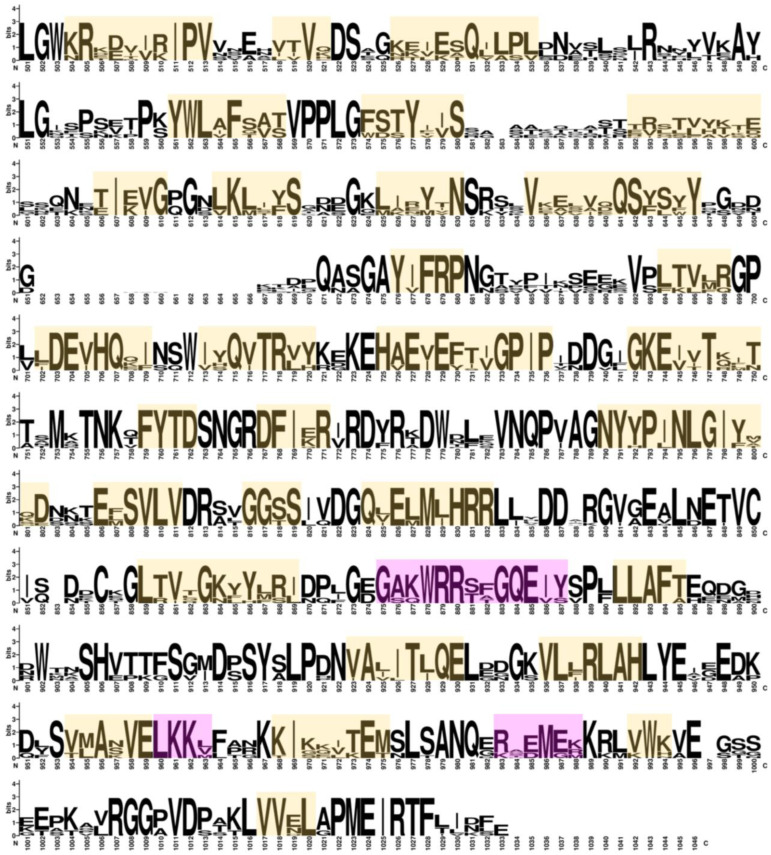
Graphic representation of the amino acid sequences of the α-man sequences found in the *Fragaria* × *ananassa* genome, summarized in a logo [26]. Bigger letters represent the conserved amino acids in the alignment. The secondary structure is represented in peach (β-strand) and pink (α-helix). Signal peptides are highlighted in light blue. The amino acids of the active and catalytic sites are signaled by a red arrow and green highlighting, respectively. The dimer interface region is inside a blue box. A black line under the sequence indicates the GH38 domain family.

A search for gene sequences encoding α-man in the *F.* × *ananassa* genome yielded 21 hits. After applying filters based on the e-value, sequence length, and redundancy, 10 sequences were obtained. Regarding the annotated terms, all sequences were associated with the biological mannose metabolic process, and their molecular functions included α-mannosidase activity [24] (Table 1). The results were validated using the Conserved Domains Database to confirm domains, and conserved amino acids at the active and catalytic sites were found in all sequences (Figure 1). Regarding the gene structure, *FaMAN7* and *FaMAN9* had 25 exons, while the other sequences had 29 exons. The open reading frame (ORF) of all *FaMAN* sequences was between 3024 and 3069 bp, and they had a protein length of 1008 to 1023 amino acids (Table 1). In *Solanum esculentum*, an α-man gene of 30 exons has been described with an ORF of 3084 bp and a 1028 amino acid sequence length [25]. The difference in exon numbers did not result in significant protein length variations (Table 1). The 76 sequences employed in the construction of the phylogenetic tree of α-man had lengths between 980 and 1047 (Supplementary Figure S1). This suggests that these sequences are near the average length of α-man proteins. Compared with other organisms, its protein length is within the typical length described in plants.

#### 2.1.2. Phylogenetic Classification of α-Man Protein Sequences

**Figure 2:**
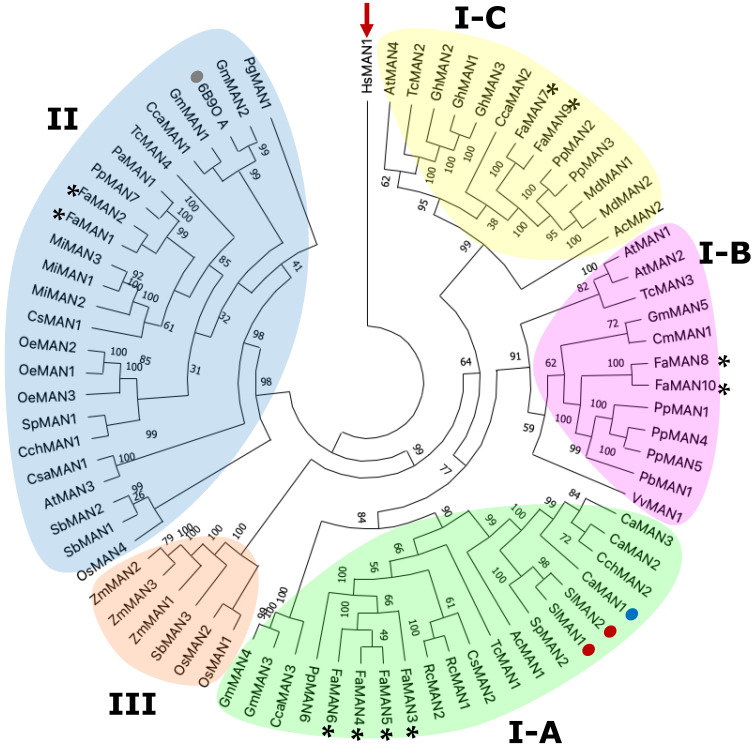
Phylogenetic tree of 76 sequences of α-man from different plant species, where sequences from *Fragaria* × *ananassa* were located in the I-A, I-B, I-C, and II groups and are tagged with a black asterisk. The sequence of *Homo sapiens* was used as an outgroup and is tagged with a red arrow. The sequences with activity assays from fruit proteins in research articles in *Solanum lycopersicum* are marked with a red dot, and those from *Capsicum annuum* are marked with a blue dot. A gray dot indicates the only sequence from a crystallographic structure reported of α-man from plants (*C. ensiformis*, PDB ID: 6B9P).

Hossain et al. [25] conducted the first phylogenetic analysis of α-man from plants, insects, and animals, and grouped 14 sequences from *Oryza sativa*, *Ricinus communis*, and *Arabidopsis thaliana* into two main groups, with one group further subdivided into three subgroups. In our study of 76 sequences, we identified three primary groups: I, II, and III (Figure 2 and Supplementary Table S1). Group I was further divided into subgroups I-A, I-B, and I-C. Subgroup I-A included *F.* × *ananassa* FaMAN3–FaMAN6, along with other sequences associated with a loss of firmness [16,17,27]. Subgroup I-B consisted of FaMAN8 and FaMAN10, which exhibited identical lengths and a 99.90% identity (Supplementary Table S2), with a slight difference in molecular weight of 0.03 kDA. Subgroup I-C, similar to I-A and I-B, comprised FaMAN7 and FaMAN9. Group II contained FaMAN1 and FaMAN2. Additionally, we observed a distinct group III in trees, primarily comprising *Poaceae* spp. However, only the sequences from *Poaceae* spp. were classified in this clade.

The clustering of *F.* × *ananassa* sequences can be attributed to its octoploidy, with sequences in subgroup I-A originating from chromosome 5 copies, as in groups I-B and I-C (copies from chromosomes 6 and 1, respectively). Tandem gene duplication between *FaMAN3* and *FaMAN4*, *FaMAN7* and *FaMAN8*, and *FaMAN9* and *FaMAN10* was suggested to be due to the differences in 442, 561, and 578 base pairs within the genome between each pair of sequences. According to Panchy et al. [28], gene duplication plays a crucial role in the genetic evolutionary novelty that is essential to adaptation. This phenomenon is common in plants, including *Arabidopsis thaliana* (17%), *Oryza sativa* (14%), *Populus trichocarpa* (16%), and *Zea mays* (35%) [29].

The translated amino acid sequences of the aforementioned gene pairs exhibited identities of 81.10, 70.01, and 69.91%, with similarities of 89, 83, and 83%, respectively. FaMAN1 and FaMAN2 shared a high identity of 98.31% in their protein sequences, but their identity with other *F.* × *ananassa* sequences was no more than 61% (Supplementary Table S2). The differences between FaMAN1 and FaMAN2, located in group II, and sequences in groups I-A to I-C, mainly involved the insertions and deletions found in loops.

#### 2.1.3. Promoter Analysis of *FaMAN* Genes

**Figure 3:**
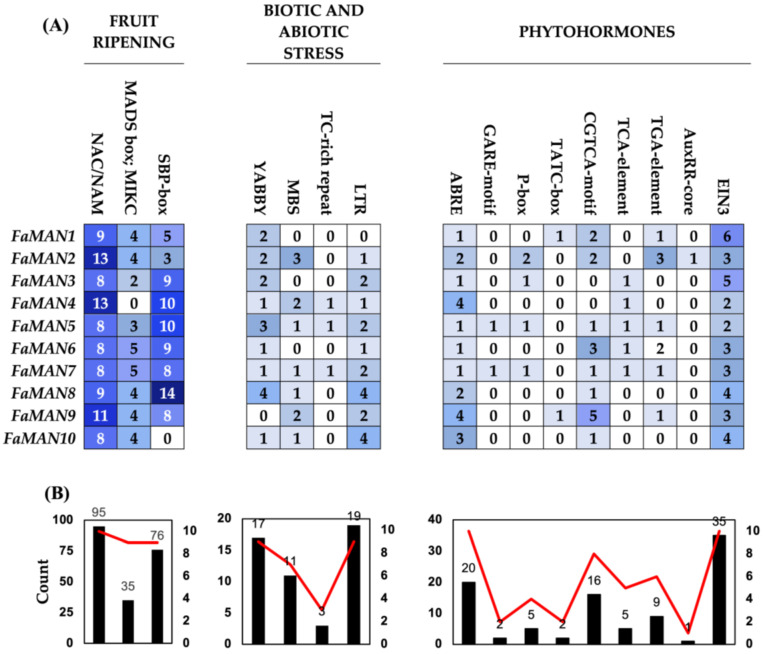
*Cis*-regulatory elements in the promoter of α-man sequences found in the *Fragaria* × *ananassa* genome. (**A**) *Cis*-regulatory element 2000 bp upstream of ATG of the ten α-mannosidase genes. The blue-to-white color changes rank in the boxes are related with a with a higher amount of *cis*-regulatory element (blue) degrading until no *cis*-regulatory element (white) is found. (**B**) Total of *cis*-regulatory elements of each (black column) and total genes with the *cis*-regulatory element (red line).

The *cis*-regulatory elements recognized by transcription factors and related to fruit ripening, NAC/NAM, MADS-box;MIKC, and SBP-box, were considered due to scientific evidence of their close relationship with the ripening process (Figure 3) [30,31,32,33]. All *FaMAN* genes were found to contain *cis*-regulatory elements to NAC/NAM, with a minimum of 8 elements and a maximum of 13 in *FaMAN2* and *FaMAN4*. Additionally, the MADS-box;MIKC *cis*-regulatory element, previously associated with fruit ripening in *Vaccinium* spp., was identified in *F.* × *ananassa*. Specifically, MADS-box elements are linked to fruit ripening processes, regulating the genes involved in auxin metabolism, abscisic acid signaling, and anthocyanin biosynthesis [34]. In tomatoes, the MADS-box acts as a positive regulator of α-mannosidase genes [27]. Among the *FaMAN* genes, only *FaMAN4* lacked a *cis*-regulatory element associated with MADS-box;MIKC. A total of 76 *cis*-regulatory elements of SBP-box were identified in *FaMAN1*–*FaMAN9*, with *FaMAN8* exhibiting the highest count of 14 *cis*-regulatory elements. SBP-box elements have been implicated in the ripening process of fruit, including tomato, banana, and loquat [35,36,37]. *Cis*-regulatory elements related to biotic and abiotic stress, including MBS, TC-rich repeats, LTR (low-temperature responsiveness), and YABBY, which are related to drought, defense, low temperature, and salt stress, have been evaluated [38,39,40,41]. YABBY and LTR are present in nine *FaMAN* genes, with 17 and 19 *cis*-regulatory elements, respectively. YABBY has been associated with salt, drought, and abscisic acid stress in different species [42], while LTR has been associated with cold tolerance in *A. thaliana*, *O. sativa*, *P. mume*, and *H. vulgare* [43,44,45]. Finally, *cis*-regulatory elements can respond to phytohormones abscisic acid (ABRE), gibberellins (GARE-motif, TATC-box, and P-box), methyl jasmonate (CGTCA-motif), salicylic acid (TCA-element), auxins (TGA-element and AUXRR), and ethylene (EIN3). *Cis*-regulatory elements that respond to abscisic acid and ethylene were found in all genes, with a total of 20 and 35 recognition sites, respectively. These *cis*-regulatory elements are crucial in the fruit ripening of climacteric and non-climacteric fruits [46]. In the case of gibberellins, although three different *cis*-regulatory elements were evaluated, not all genes were responsive to this phytohormone (*FaMAN4*, *FaMAN6*, *FaMAN8*, and *FaMAN10*). However, in *F.* × *ananassa*, gibberellins are related principally to cell division and breaking dormancy [47]. Another important phytohormone in *F.* × *ananassa* is auxin, a phytohormone present in the first fruit developmental stages [48]. A total of 10 *cis*-regulatory elements were identified between the TGA element and AUXRR. However, *FaMAN3*, *FaMAN4*, *FaMAN8*, and *FaMAN10* do not possess *cis*-regulatory elements that respond to auxins.

### 2.2. Molecular Assays

#### 2.2.1. Relative Expression of *FaMAN* Genes

**Figure 4:**
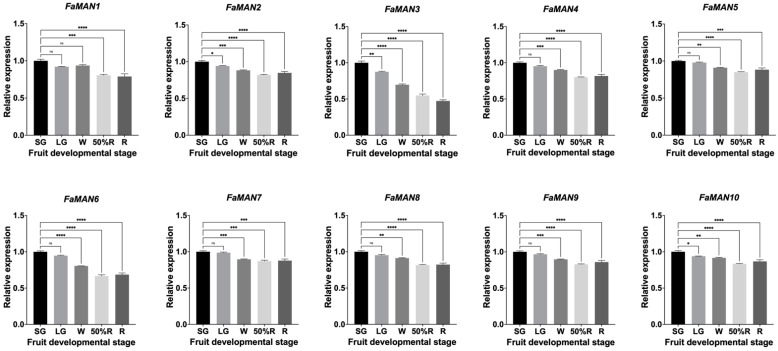
Relative expression of *FaMAN1*–*FaMAN10* genes from *Fragaria* × *ananassa* ‘Camarosa’ in different fruit developmental stages: small green (SG), large green (LG), white (W), 50% ripe (50%R), and ripe (R) (ns = *p*-value > 0.05; * = *p*-value ≤ 0.05; ** = *p*-value ≤ 0.01; *** = *p*-value ≤ 0.001; **** = *p*-value ≤ 0.0001 according to Prism software Version 10.0.3).

qPCR analysis revealed a consistent pattern in the transcriptional levels of the ten α-man genes. There were high levels of transcripts in the SG stage, which decreased in the 50%R stages for *FaMAN2* and *FaMAN4* to *FaMAN10*, and increased in the R stage (Figure 4). *FaMAN1* showed a decrease in the LG stage, followed by an increase in the W stage, and then a decrease again in the R stage. *FaMAN3* was the only gene that showed a consistent decrease in expression across all fruit ripening stages (Figure 4).

In the study by Ghosh et al. [17] on α-man genes from *C. annum* (GenBank ID: GU356594), a decrease in transcript levels was observed from the S4 to S6 fruit ripening stages, which coincided with the growth (S4 to S5) and changes in color (S5). However, in the ripe stage (S6), there was a significant increase in the relative expression of this α-man gene, suggesting its participation in the fruit ripening and softening of *C. annum*. Similarly, in the fruit ripening of *S. lycopersicum* (Solgenomics ID: mRNA Solyc06g068860.2.1), the mature green stage presented low levels of the relative expression of α-man compared to the other ripening stages. The highest transcription levels were found in the breaker ripe stage, followed by a gradual decrease until the ripe stage [27].

In *Prunus salicina* L., the relative expression post-harvest increased in ‘Early Golden’, which is characterized by early ripening and a short shelf life [19]. However, in ‘V98041’, which is a late-ripening and long-shelf-life fruit, the post-harvest increase in relative expression occurred to a lesser extent compared to ‘Early Golden’. Hormonal treatments involving auxin and ethylene revealed three groups of α-man genes: (1) genes insensitive to auxin treatments; (2) genes with auxin dependence, and (3) genes responsive to both auxin and ethylene treatment [19]. In *F.* × *ananassa*, it has been observed that auxin levels decrease at the onset of fruit ripening, followed by an increase in abscisic acid levels [48,49]. Ethylene has been linked to normal fruit development and can influence color, firmness, and aroma [50]. Although hormonal treatments were not conducted in this research, analysis of the promoter regions of the 10 *FaMAN* genes revealed that *FaMAN3*, *FaMAN4*, *FaMAN8*, and *FaMAN10* lack a *cis*-regulatory element for auxins (Figure 3A). Despite the suggestion in the literature that ethylene may not play a significant role in *F.* × *ananassa* ripening, all genes contained *cis*-regulatory elements for ethylene (Figure 3B).

#### 2.2.2. Total α-Man Enzyme Activity in Fruit Ripening

**Figure 5:**
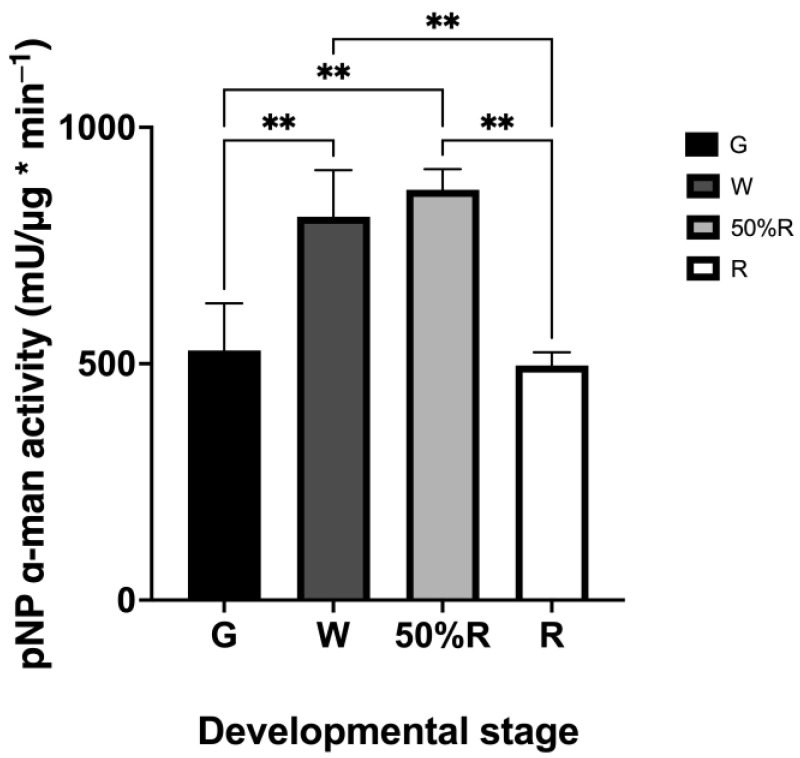
Enzyme activity of total α-man in *F.* × *ananassa* in different fruit developmental stages: green (G), white (W), 50% ripe (50%R), and ripe (R). No significant results in the statistical analysis were omitted (** = *p*-value ≤ 0.01, according to Prism software Version 10.0.3).

Enzyme activity assays were conducted on the total proteins extracted from *F.* × *ananassa* at four developmental stages (Figure 5). The G stage was considered the average of the SG and LG stages, as both precede ripening events and because these stages are related to fruit development. No significant differences were observed between the G and R stages or between the W and 50%R stages, with the highest activity observed in the W and 50%R stages. The other stages showed significant differences in their measurements (*p*-value = 0.005). This could be associated with an increase in the production of cell wall proteins, where up to 50%R of total proteins may undergo N-glycosylation [1]. Many N-glycosylation structures in plants are enriched with mannose glycans, suggesting that the molecular machinery may prioritize cutting the glycosidic bond of mannose over other sugars in N-glycosylations, such as galactose or fucose. Enzyme activity assays were realized in climacteric and non-climacteric fruits such as *S. lycopersicum*, *P. communis*, *Prunus persica*, and *P. salicina.* A reduction in enzyme activity was observed [2,16,19,20]. There does not appear to be a direct correlation between the dependence on phytohormones for ripening and an increase or decrease in α-man activity. We hypothesize that activity levels may be determined by other molecular conditions, and for this reason, it would be of value to study the N-glycan biosynthesis pathway.

Regarding the evidence of α-man enzyme activity in other fruits, the following has been reported. In relation to N-glycosylations and organoleptic changes in fruits in melting peaches, proteins were analyzed from 80 days after full bloom to 7 days after harvest, revealing an increase in enzyme activity [2]. This suggests a cascade of molecular events occurring after fruit collection. In *S. lycopersicum*, high levels of α-man enzyme activity have been reported at the breaker stage, coinciding with the degradation of the green color [16,27]. The optimum conditions for α-man activity isolated from ripe *Lycopersicum esculentum* were described by Hossain et al. [51], with a pH of 5.5 and a temperature of 40 °C identified. In α-man isolated from *C. annum*, a non-climacteric fruit, α-man transcript levels were correlated with enzyme activity, with duplication observed at the S6 stage. The optimum conditions were a pH of 6.0 and a temperature of 55 °C [17]. α-man enzyme activity can be inhibited by alkaloids, such as swainsonine, 1-deoxy-mannojirimycin, and 1-deoxynojirimycin [51,52].

## 3. Materials and Methods

### 3.1. Identification of FaMAN Genes

Beginning with the GenBank sequence ID EU244853, as published by Meli et al. [16], a sequence hunt for α-man was conducted within the Genome Database for Rosaceae (GDR), utilizing the *Fragaria* × *ananassa* ‘Camarosa’ Genome v1.0.a2 database (re-annotation of v1.0.a1) [53]. Non-repetitive sequences with an e-value of zero and a sequence length exceeding 1000 amino acids were chosen for further analysis. The molecular weight and isoelectric point of all sequences were evaluated with webserver Compute pI/Mw from Expasy (https://www.expasy.org/). ORF and protein length, gene ID, and chromosomal location and strand were obtained from GDR.

### 3.2. In Silico Analysis of Promoter Sequences

For every obtained candidate sequence, the promoter region was scrutinized by extracting 2000 base pairs upstream of the 5′ UTR region. Each promoter underwent analysis using the PlantCARE and PlantPan 3.0 databases [54,55]. *Cis*-regulatory elements associated with responses to fruit ripening, phytohormones, and biotic and abiotic stressors were annotated. For the PlantPan database, transcription factors from *A. thaliana*, *Brachypodium distachyon*, *Glycine max*, *Malus domestica*, *O. sativa*, *Sorghum bicolor*, and *Zea mays* were specifically selected.

### 3.3. Phylogenetic Analysis of the FaMAN Enzyme Family

In the protein analysis, the conserved domains of each sequence were assessed using NCBI’s Conserved Domains Database web server [56]. To elucidate the phylogenetic classification of the α-man enzyme through a phylogenetic tree, sequence alignment was conducted using the Clustal Omega web server [57]. Subsequently, to establish a standardized classification for any plant α-man, sequences were retrieved from the protein NCBI Database using the keywords “alpha-mannosidase” and “GH38”. Sequences were filtered by species “Plants” and a length between 1000 and 1200 residues. Sequences with headers labeled as ‘partial’, ‘like’, and ‘low quality’ were excluded from the alignment. The downloaded sequences from NCBI were aligned using Clustal Omega. Both alignments utilized the neighbor-joining algorithm with 10,000 bootstrap iterations to construct the phylogenetic tree using MEGA11 [58].

### 3.4. Fragaria × ananassa Harvest in Orchard

*Fragaria* × *ananassa* ‘Camarosa’ was collected in 4 developmental stages, classified as small green (SG), white (W), 50% ripe (50%R), and ripe (R), according to Ramos et al. [23]. A total of 30 fruits by developmental stage were collected in the spring of 2022, specifically in the morning of October 26 (between 8:00 and 9:30 a.m.). The fruit was obtained from a commercial orchard located in Chanco, Séptima Region del Maule, Chile (35°44′03.3″ S, 72°31′59.6″ W). The collected fruit was immediately transported to the Multidisciplinary Agroindustry Research Laboratory of the Universidad Autónoma de Chile in Talca, Region del Maule (35°25′9.259″ S, 71°40′11.183″ W), disinfected with sodium hypochlorite (0.05%), and stored at −80 °C until later use.

### 3.5. RNA Extraction, cDNA Synthesis, and Real-Time qPCR (RT-qPCR) Assays

The RNA extraction from samples from all developmental stages was realized using an RNA extraction kit (PureLink™ RNA Min, Invitrogen, Carlsbad, CA, USA), following the manufacturer’s instructions. After total RNA extraction, DNase treatment was used to remove genomic DNA contamination and cDNA was synthesized with a RevertAid RT kit (Thermo Fisher Scientific, Vilnius, Lithuania), according to the manufacturer’s protocol. For RT-qPCR, primers were designed using the 5′ UTR region (Table 2). For each primer pair, the reaction and quantification were undertaken according to the protocol described in Ramos et al. [23], using the expression level of *F.* × *ananassa* glyceraldehyde-3-phosphate-dehydrogenase 1 (*FaGAPDH1*) as a normalizer gene. The experiment was carried out using the AriaMx Real-Time PCR System (Agilent Technologies Inc. Santa Clara, CA, USA). Three biological and two technical replicates were implemented per total RNA extraction per developmental stage. The results were evaluated using the CT Pfaffl method [59]. A one-way analysis of variance (ANOVA) with Dunnett’s multiple comparison test was carried out using Prism 10 Software (GraphPad Software, San Diego, CA, USA). Statistically significant differences were considered at a threshold of *p*-value ≤ 0.05.

### 3.6. Total Protein Extraction and Enzymatic Activity Assay for α-Man

Protein extraction was performed following the methods outlined in Bose et al. [22] and Jagadeesh et al. [60], with some modifications. Two grams from a pool of frozen *F.* × *ananassa* fruit was ground with liquid nitrogen. Subsequently, each extract was mixed with 10 mL of sodium acetate buffer (100 mM, pH 5.0) containing 1 mM phenylmethanesulfonyl fluoride and 0.5% polyvinylpyrrolidone, followed by overnight agitation. The supernatant was centrifuged at 10,000× *g* for 15 min at 4 °C. The protein concentration was increased using a protein concentrator PES (Thermo Fisher Scientific, Rockford, IL, USA) with a 30 K > MWCO. The protein concentration was determined using the Bradford method, utilizing a bovine serum albumin standard calibration curve (Roche, Germany) [61]. The total enzymatic activity of α-man was assessed according to previously described protocols [22,60], with modifications. As a substrate, 4-nitrophenyl-α-D-mannopyranoside (*p*NP-Man; Gold Biotechonology^®^ San Luis, MI, USA) was prepared at 1.0 mM in distilled water. The enzymatic activity assay involved three technical replicates per bulk of sample. We incubated the sodium acetate buffer (100 mM pH 5.0) with 100 µL of the *p*NP-man substrate at 37 °C for 5 min. Then, the crude extract of the total fruit proteins was added to the mixture in an Eppendorf tube, and the reaction was incubated at 37 °C for 15 min with soft mixing every 5 min. The reaction was terminated by adding 500 mM of Na_2_CO_3_. After 5 min, the absorbance at 410 nm was measured using an Epoch 2 Microplate Spectrophotometer (BioTek^®^, Santa Clara, CA, USA) and analyzed using Gen5 V2.09 software. Statistical analysis was carried out with Prism 10 Software (GraphPad Software, San Diego, CA, USA). A one-way ANOVA and Tukey’s multiple comparison test were implemented to evaluate the results using a *p*-value ≤ 0.01, according to Prism software Version 10.0.3.

## 4. Conclusions

In *F.* × *ananassa*, vesicular α-man is involved in the early stages of ripening, where the relative expression is higher in SG and decreases until the R stage. For the total enzyme activity of the fruit, we observed an increase in activity in the W and 50%R stages. The G and R stages showed similar activity levels. These findings may be related to N-glycosylation structures, which, in plants, have been described as high in mannose (oligomannose), complex, hybrid, and paucimannose, and all of them have at least mannose and N-acetylglucosamine in their composition [62]. Structures with an increased amount of mannose and mixed N-glycosylation structures in *F.* × *ananassa* could be more abundant in the W and 50%R stages. *Cis*-regulatory elements that respond to ripening were found to be abundant in the 10 genes. Responses to phytohormones that included negative or positive regulation of the relative expression of *FaMAN* genes were observed. Accordingly, phytohormone or inhibitor applications could help detect their effect on the expression of *FaMAN* genes. This could be useful as an agronomic strategy to prolong shelf life through the inhibition of N-glycans in fruits.

## Figures and Tables

**Table 1:** Summary of the coding of 10 α-man genes found in the *Fragaria* × *ananassa* genome.

Gene Name	Gene ID	Chromosomal Location	Strand	ORF Length (bp)	Protein Length (aa)	*p*I Value	MW (kDA)
*FaMAN1*	FxaC_2g16900.t2	Fvb1-2:7637155..7644785−	−	3024	1008	5.68	112.94
*FaMAN2*	FxaC_1g12790.t1	Fvb1-4:5491201..5498753−	−	3024	1008	5.81	113.22
*FaMAN3*	FxaC_17g11250.t1	Fvb5-1:5272631..5279568−	−	3066	1022	6.28	115.27
*FaMAN4*	FxaC_17g11260.t1	Fvb5-1:5280010..5286714−	−	3033	1011	5.70	113.69
*FaMAN5*	FxaC_20g09800.t1	Fvb5-2:5096401..5103273−	−	3033	1011	5.66	113.92
*FaMAN6*	FxaC_19g09610.t1	Fvb5-4:4739310..4746318−	−	3024	1008	5.70	113.54
*FaMAN7*	FxaC_21g01480.t1	Fvb6-1:668217..675394+	+	3048	1016	5.74	113.99
*FaMAN8*	FxaC_21g01481.t1	Fvb6-1:675955..685254+	+	3069	1023	5.82	115.45
*FaMAN9*	FxaC_23g44241.t1	Fvb6-2:27453374..27460546−	−	3048	1016	5.84	113.84
*FaMAN10*	FxaC_23g44240.t1	Fvb6-2:27444620..27452796−	−	3069	1023	5.82	115.42

ORF: Open reading frame.

**Table 2:** Primer sequences (5′ → 3′) of α-man genes for qPCR experiments.

Name	Forward Primer Sequence	Reverse Primer Sequence
*FaMAN1*	GGACGTTCCCTTCTCTCTCTATAA	CATTTCCACACATGAAACGACCA
*FaMAN2*	CTTTGACGCAAGCACCACACAATG	CACACACTAAACGACCAAAAACTGAAGCA
*FaMAN3*	GTTAACAACGAAATGCTGTAGCAC	ATGGAAACACAACTAGTACCATAAGAAGC
*FaMAN4*	GTATCAATCAATTAATCGACAAAGACAACG	CAACTGCCATTGAAATGCAGGAG
*FaMAN5*	GCTAGTATCAATTAATCGACATAGACAACG	ACGTCGTATTGTACTGTATGTACTTGG
*FaMAN6*	ACCAATGCACACCTAGCTAGTAT	AGTAAGCCATTGAAATGCGTGAGT
*FaMAN7*	TCCATTTCTTCGATCCTTCGTTTTTGG	CCATAGCTGAAAGCTTCACTGAAACT
*FaMAN8*	AGAAGTCAGACAGAAAAAACAGTACAAG	AAGAAGAGCCAAGAAGAAGAGTAAACC
*FaMAN9*	CTTCGATCCTTCGTTTTTGGCTTCTTATG	CAGTAACACTACCAGCAACAGCAACG
*FaMAN10*	GCATTAGGAAGCGGAAGAAGAATG	CTTCTTAGCTACCTTATTGCTTTGCTT
*FaGAPDH1*	TCCATCACTGCCACCCAGAAGACTG	AGCAGGCAGAACCTTTCCGACAG

## Data Availability

Data contained within the article.

## Supplementary Materials

The following supporting information can be downloaded at: https://www.mdpi.com/article/10.3390/ijms25126581/s1. References [63,64,65,66,67,68,69,70,71,72,73,74,75,76] are cited in the Supplementary Materials.
