# Supplementary material for: Involvement of the GH38 Family Exoglycosidase α-Mannosidase in Strawberry Fruit Ripening
Source: Int J Mol Sci. 2024 Jun 14;25(12):6581. doi: 10.3390/ijms25126581 (PMC11203768; doi:10.3390/ijms25126581)
Supplement: Supplementary file 1 [file ijms-25-06581-s001.zip › Table S1.pdf]

Table S1. Specific information about the 76 sequences employed to build the phylogenetic three of the Figure 2.

Scientific name	Accesion ID	Assigned name	Protein length (aa)	Reference
Actinidia chinensis	PSR95975.1	AcMAN1	1021	-
Actinidia chinensis	PSR87445.1	AcMAN2	1015	-
Arabidopsis thaliana	NP_196902.2	AtMAN1	1024	[64]
Arabidopsis thaliana	BAB11126.1	AtMAN2	1030	[65]
Arabidopsis thaliana	NP_201416.1	AtMAN3	1047	[64]
Arabidopsis thaliana	NP_189306.1	AtMAN4	1019	[66]
Cajanus cajan	KYP66601.1	CcaMAN1	1015	[67]
Cajanus cajan	KYP41565.1	CcaMAN2	1015	[67]
Cajanus cajan	KYP37037.1	CcaMAN3	1026	[67]
Canavalia ensiformis	6B9O_A	CeMAN1	980	[68]
Capsicum annuum	ADU20406.1	CaMAN1	1030	[17]
Capsicum annuum	KAF3641428.1	CaMAN2	1025	-
Capsicum annuum	KAF3623492.1	CaMAN3	1025	-
Capsicum chinense	PHU26937.1	CchMAN1	1006	[69]
Capsicum chinense	PHU15266.1	CchMAN2	1041	[69]
Citrus sinensis	XP_006466974.1	CsMAN1	1004	-
Citrus sinensis	XP_015387782.1	CsMAN2	1010	-
Cucumis melo	AGX24942.1	CmMAN1	1020	-
Cucumis sativus	XP_004135022.1	CsaMAN1	1007	-
Fragaria x ananassa	-	FaMAN1	1007	This paper
Fragaria x ananassa	-	FaMAN2	1007	This paper
Fragaria x ananassa	-	FaMAN3	1021	This paper
Fragaria x ananassa	-	FaMAN4	1010	This paper
Fragaria x ananassa	-	FaMAN5	1010	This paper
Fragaria x ananassa	-	FaMAN6	1007	This paper

Fragaria x ananassa	-	FaMAN7	1015	This paper
Fragaria x ananassa	-	FaMAN8	1022	This paper
Fragaria x ananassa	-	FaMAN9	1015	This paper
Fragaria x ananassa	-	FaMAN10	1022	This paper
Glycine max	KAH1266875.1	GmMAN1	1014	[70]
Glycine max	KAH1260237.1	GmMAN2	1012	[70]
Glycine max	KAH1255207.1	GmMAN3	1015	[70]
Glycine max	KAH1245996.1	GmMAN4	1022	[70]
Glycine max	KAH1204907.1	GmMAN5	1030	[70]
Gossypium hirsutum	XP_040933499.1	GhMAN1	1029	-
Gossypium hirsutum	XP_016696014.1	GhMAN2	1022	-
Gossypium hirsutum	XP_016668043.1	GhMAN3	1022	-
Homo sapiens	AAC34130.1	HsMAN1	1011	[71]
Malus x domestica	XP_028963445.1	MdMAN1	1023	-
Malus x domestica	XP_008379047.2	MdMAN2	1021	-
Mangifera indica	XP_044500627.1	MiMAN1	1003	-
Mangifera indica	XP_044500618.1	MiMAN2	1004	-
Mangifera indica	XP_044500612.1	MiMAN3	1004	-
Olea europea	CAA2983802.1	OeMAN1	1010	-
Olea europea	CAA2971903.1	OeMAN2	1014	-
Olea europea	CAA2935151.1	OeMAN3	1001	-
Oryza sativa	ABG22500.1	OsMAN1	1018	[72]
Oryza sativa	EEC68272.1	OsMAN2	1022	[73]
Oryza sativa	EEE50554.1	OsMAN4	1004	-
Prunus avium	XP_021816006.1	PaMAN1	1015	-
Prunus persica	AGC10269	PpMAN1	1024	-
Prunus persica	ONI20072.1	PpMAN2	1029	[74]

Prunus persica	ONI20071.1	PpMAN3	1027	[74]
Prunus persica	ONI20068.1	PpMAN4	1024	[74]
Prunus persica	ONI20067.1	PpMAN5	1024	[74]
Prunus persica	ONI07846.1	PpMAN6	1014	[74]
Prunus persica	ONH97113.1	PpMAN7	1015	[74]
Punica granatum	XP_031387946.1	PgMAN1	1010	-
Pyrus x bretschneideri	AGR44468.1	PbMAN1	1024	-
Ricinus communis	EEF51696.1	RcMAN1	1012	-
Ricinus communis	XP_002511094.1	RcMAN2	1012	-
Solanum lycopersicum	NP_001234851.1	SlMAN1	1028	[27]
Solanum lycopersicum	ABY83271.1	SlMAN2	1029	[16]
Solanum pennellii	XP_015064110.1	SpMAN1	1009	-
Solanum pennellii	XP_015079090.1	SpMAN2	1029	-
Sorghum bicolor	XP_021305978.1	SbMAN1	1011	-
Sorghum bicolor	XP_002464754.2	SbMAN2	1017	-
Sorghum bicolor	XP_002449583.1	SbMAN3	1019	-
Theobroma cacao	EOY22633.1	TcMAN1	1010	[75]
Theobroma cacao	EOY02952.1	TcMAN2	1015	[75]
Theobroma cacao	EOY02951.1	TcMAN3	1018	[75]
Theobroma cacao	EOX90816.1	TcMAN4	1012	[75]
Vitis vinifera	XP_002276092.2	VvMAN1	1025	-
Zea mays	PWZ38051.1	ZmMAN1	1016	[76]
Zea mays	ONM26645.1	ZmMAN2	1018	-
Zea mays	ONM26644.1	ZmMAN3	1016	-
