# Supplementary material for: Involvement of the GH38 Family Exoglycosidase α-Mannosidase in Strawberry Fruit Ripening
Source: Int J Mol Sci. 2024 Jun 14;25(12):6581. doi: 10.3390/ijms25126581 (PMC11203768; doi:10.3390/ijms25126581)
Supplement: Supplementary file 1 [file ijms-25-06581-s001.zip › Table S2.pdf]

Table S2. Percentage of identity between FaMANs proteins from *F. x ananassa*.

	FaMAN1	FaMAN2	FaMAN3	FaMAN4	FaMAN5	FaMAN6	FaMAN7	FaMAN8	FaMAN9	FaMAN10
**FaMAN1**	-									
**FaMAN2**	98.31	-								
**FaMAN3**	58.50	58.92	-							
**FaMAN4**	60.47	60.14	81.10	-						
**FaMAN5**	60.47	60.14	81.24	98.81	-					
**FaMAN6**	60.57	60.34	81.54	99.01	98.91	-				
**FaMAN7**	59.82	59.92	68.86	70.75	70.75	70.65	-			
**FaMAN8**	60.32	60.12	69.79	73.94	73.54	73.74	70.01	-		
**FaMAN9**	59.62	59.72	68.66	70.55	70.55	70.45	99.70	69.95	-	
**FaMAN10**	60.32	60.12	69.79	73.94	73.55	73.74	70.01	99.90	69.91	-
