# Supplementary figures and images for: Involvement of the GH38 Family Exoglycosidase α-Mannosidase in Strawberry Fruit Ripening
Source: Int J Mol Sci. 2024 Jun 14;25(12):6581. doi: 10.3390/ijms25126581 (PMC11203768; doi:10.3390/ijms25126581)

**Figure S1.** Structure of *FaMAN* genes. Figure elaborated with GSDS 2.0 [63].

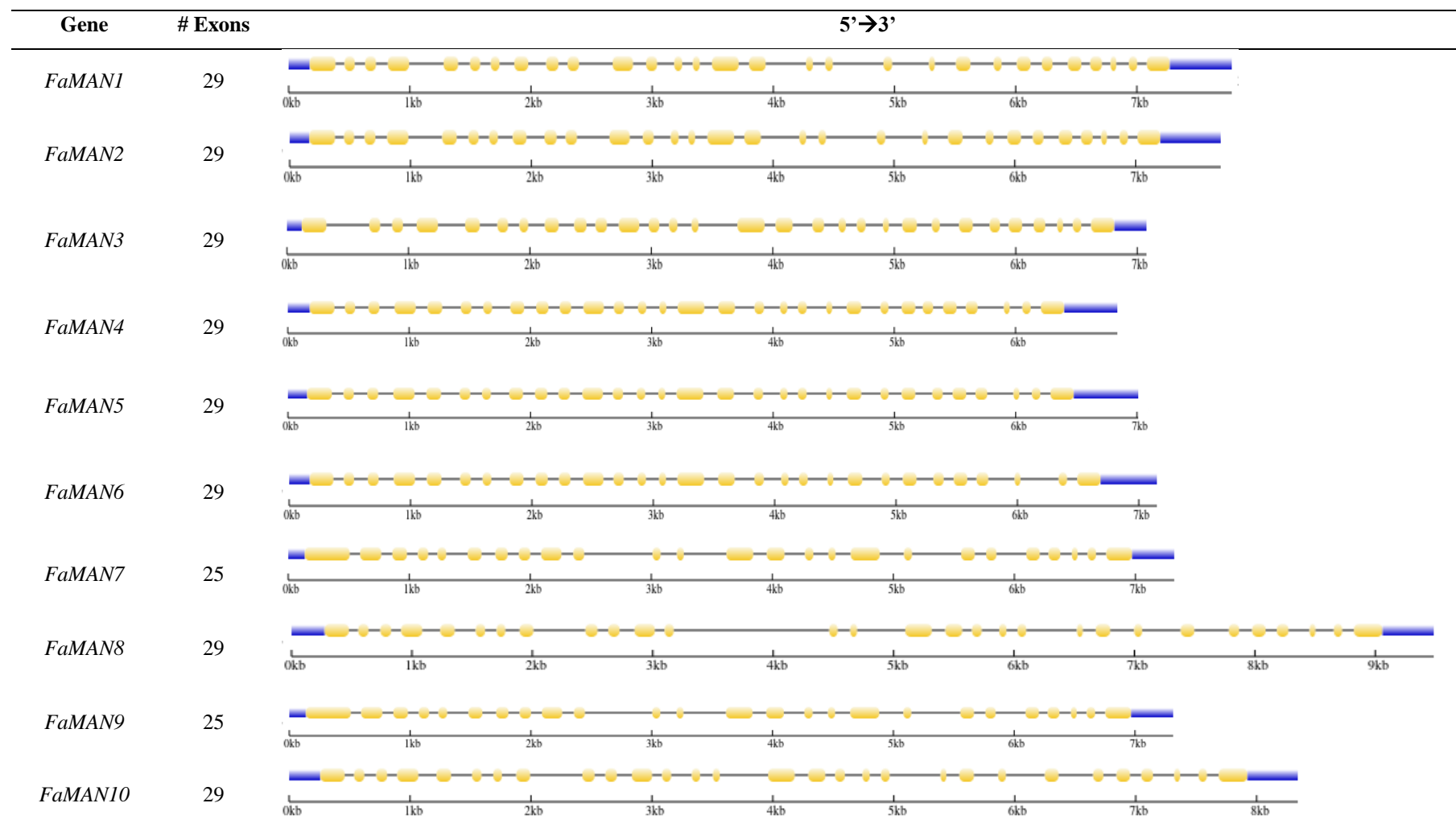

Supplement: Supplementary file 1 [file ijms-25-06581-s001.zip › Figure S1.pdf]
